# Serum paraprotein persistence and size determine outcome in a cohort of patients with a modern definition of plasmacytoma with up to 19 years of follow up

**DOI:** 10.1038/s41408-021-00419-1

**Published:** 2021-02-08

**Authors:** Elisabet E. Manasanch, Chutima Kunacheewa, Catherine M. Claussen, Hans C. Lee, Sheeba K. Thomas, Jill Gunther, Chelsea C. Pinnix, Bouthaina S. Dabaja, Behrang Amini, Raymond Alexanian, Robert Z. Orlowski, Wenli Dong, Lei Feng, Donna M. Weber

**Affiliations:** 1 Department of Lymphoma/Myeloma, The University of Texas MD Anderson Cancer Center, Houston, TX USA; 2 Faculty of Medicine Siriraj Hospital, Division of Hematology, Mahidol University, Salaya, Thailand; 3 Department of Radiation Oncology, The University of Texas MD Anderson Cancer Center, Houston, TX USA; 4 Department of Radiodiagnostic Imaging, The University of Texas MD Anderson Cancer Center, Houston, TX USA; 5 Department of Biostatistics, The University of Texas MD Anderson Cancer Center, Houston, TX USA

**Keywords:** Myeloma, Clinical trials

Dear Editor,

Solitary plasmacytoma (SP) is a rare plasma cell disorder (<5% of all plasma cell dyscrasias) that consists of solitary bone plasmacytoma (SBP) and solitary extramedullary plasmacytoma (SEP). Local control with radiation therapy (RT) remains the standard of care. About half of all patients progress to multiple myeloma (MM) within 3–5 years after diagnosis^1^. Several factors identify SP patients that are at high risk of progression to MM (location, bone marrow (BM) involvement, serum free light chain values (sFLC), paraprotein persistence, and size). Identification of patients at high risk of progression to MM is an important step to design prevention strategies. In smoldering myeloma, randomized studies have shown increased progression-free survival (PFS) and overall survival (OS) after systemic treatment^2,3^ and immunotherapeutic strategies in this space are promising^4,5^. Due to a limited number of cases, prospective evaluation of prevention strategies in SP has remained elusive.

We retrospectively reviewed medical records of patients treated between 1986 and 2015 in our institution’s database. Patients met diagnostic criteria of SP according to the International Myeloma Working Group 2014^6^. 218 patients were identified, and 147 excluded (Fig. S1). At baseline, patients were evaluated to exclude systemic involvement by^1^ bone marrow biopsy to evaluate plasma cell involvement by IHC and at least 4-color flow cytometry (FC) including CD138, CD38, CD19, CD56, and CD33^2^; skeletal survey and advanced imaging –computed tomography (CT), magnetic resonance imaging (MRI), and/or positron emission tomography/CT (PETCT). Development of either a new SP outside the area of radiation, multiple plasmacytomas, or SLiM CRAB criteria attributed to underlying plasma cell dyscrasia was considered progression to MM.

The primary objective of the study is to describe a model with variables that affect progression from SP to MM. Additionally, we aimed to assess the time to myeloma progression (TTM: time from diagnosis to myeloma progression), PFS (time from diagnosis to myeloma progression or death), and OS (time from diagnosis to death from any cause). *χ*^2^ test and Fisher’s exact test were used to evaluate the association between categorical variables. Wilcoxon’s rank sum test was used to compare continuous variables between two different groups. TTM, PFS, and OS were evaluated by Kaplan–Meier curves and log-rank test. In the process of fitting the multi-covariate Cox regression model, a backwards variable selection was used with a *p*-value ≤0.05 as the limit for inclusion in the model for predicting risk of progression to MM and a *p*-value threshold of 0.2 for PFS and OS.

Seventy-one patients (50 SBP and 21 SEP) met inclusion criteria (Table 1). At median follow-up of 9.4 years (0.3–19.5 years), 35 patients progressed to MM, 28/50 (56%) patient in SBP and 7/21 (33%) patients in SEP (*p* = 0.083; Fig. S2). In all, 3-, 5-, and 10-year TTM rate were 34%, 38%, and 50% for the entire cohort (Table S1). The median TTM for the entire patient cohort was not reached (NR) and was shorter in SBP patients (6.7 years) than SEP patients (NR; *p* = 0.138). All SEP patients who progressed to myeloma did so within 5 years of diagnosis. Similar results were reported that showed that 84% of patients with SEP progressed within the first 3 years of the diagnosis^7^. In contrast, in our series, SBP patients continued to progress after 14 years of diagnosis.

**Table 1:** Baseline patients’ characteristics and treatment profiles.

	All, *n* (%)	SBP, *n* (%)	SEP, *n* (%)	*P*-value
	*n* = 71	*n* = 50	*n* = 21	
Age, median (min, max)	58 (32–83)	57 (36–83)	61 (32–75)	0.588
Sex, male	51 (70)	31 (62)	20 (95)	0.005
Imaging^a^				NA
PET/CT	26 (37)	20 (40)	6 (29)	
MRI	63 (88)	47 (94)	16 (76)	
CT	44 (62)	29 (58)	15 (71)	
Bone survey	38 (53)	39 (78)	16 (76)	
Advance imaging^b^	71 (100)	50 (100)	21 (100)	
M-protein, positive	30 (46)	26 (58)	4 (19)	0.003
M-protein, level; median (min, max)	0 (0–2)	0.3 (0–2)	0 (0–0.6)	0.006
M-protein >0.7 g/dL	13 (19)	12 (25)	1 (5)	0.05
Serum IFE, positive	67 (94)	47 (94)	20 (95)	0.838
sFLC ratio (involved/uninvolved) (*n* = 43)	2.3 (1.1– 148)	2.3 (1.1– 148)	1.3 (1.1–3.4)	0.119
Urine IFE, positive (*n* = 64)	26 (40)	20 (44)	6 (32)	0.342
Bence Jones Protein, positive (*n* = 66)	21 (32)	17 (37)	4 (20)	0.295
Occult marrow disease^c^, positive by MFC	7 (10)	6 (12)	1 (5)	0.345
Location				<0.001
Head and neck	21 (30)	2 (4)	19 (91)	
Spine	15 (21)	15 (30)	0	–
Pelvis	10 (14)	10 (20)	0	–
Lower extremities	7 (10)	7 (14)	0	–
Upper extremities	7 (10)	7 (14)	0	–
Chest	8 (11)	7 (14)	1 (5)	–
Abdomen	3 (4)	2 (4)	1 (5)	–
Size, median (range) >5 cm	4 (1–17)	5 (1–17)	3 (1–10)	0.087
Immunoparesis^d^ (*n* = 67)	6 (9)	4 (9)	2 (10)	0.913
LDH (IU/L), median (range)^e^ (*n* = 65)	465 (331– 818)	447 (331– 818)	481 (370– 566)	0.914
Β2 microglobulin (mg/L), median (range)	2 (1.2–4.1)	2 (1.2–3.6)	2 (1.4–4.1)	0.883
Albumin (g/dL), median (range)	4.4 (2.2–5.4)	4 (2.2–5.2)	4 (3.1–5.4)	0.709
Persistence of serum paraprotein 1 year after treatment (*n* = 61)	19 (31)	18 (41)	1 (6)	0.052
Treatment (*n* = 71)				
Radiation alone	52 (74)	42 (84)	10 (47)	–
RT + surgery	13 (19)	6 (12)	7 (33)	NA
RT + chemotherapy^f^	1 (1)	0	1 (5)	–
RT + surgery + chemotherapy^f^	1 (1)	0	1 (5)	–
Surgery alone	4 (6)	2 (4)	2 (9)	–
Radiation dose, median (range) Gy	45 (24– 50)	45 (24– 50)	45 (40– 50)	0.161

Abbreviations: *BM*, bone marrow; *CT*, computed tomography; *LDH*, lactase dehydrogenase; *MRI*, magnetic resonance imaging; *NA*, not applicable; *PET/CT*, positron emission tomography–computed tomography; *RT*, radiation therapy; *MFC*, multicolor flow cytometry; *IHC*, immunohistochemistry; *PC*, plasma cell

^a^Available at our institution since: 1986 (bone survey x-rays and CT scan), 1988 (MRI), 2005 (PETCT).

^b^Advance imaging including CT, MRI, or PET/CT

^c^Occult marrow disease was tested by MFC in 71 patients who all had ≤5% plasma cells in the bone marrow core biopsy by immunohistochemistry

^d^Immunoparesis was defined as level of ≥1 uninvolved immunoglobulin below the lower level of normal

^e^LDH institutional normal range is 135-225 IU/L

^f^Chemotherapy consisted of CHOP, Taxol, melphalan, and modified cyclophosphamide, bortezomib, Adriamycin, dexamethasone (CVAD)

Due to large number of years of follow up and retrospective nature of the study, not all patients had information for all variables. Variables with and additional (*n* =) list the number of patients for whom the variable information is available. If no additional (*n* =) is present, then that particular variable’s information is available for all 71 patients

The presence of SMP at baseline and 1 year after treatment and plasmacytoma size of ≥10 cm were associated with increased risk of progression to MM. Patients who had presence of a serum monoclonal protein (SMP) at baseline and a plasmacytoma size of ≥10 cm, compared to those who did not, had a lower median TTM of 5.3 years versus NR (*p* = 0.002), and 1.5 years versus NR (*p* = 0.033), respectively. Those patients that had persistent SMP 1 year after treatment had a shorter TTM, 1.8 years versus NR compared to those who did not (*p* < 0.001; Fig. S2).

We included age, LDH, gender, β2-microglobulin, plasmacytoma type, size, immunoparesis, SMP presence at baseline, SMP persistence at 1 year after treatment, involved/uninvolved sFLC ratio, Bence Jones proteinuria, and radiation dose in univariate analysis (Tables S1 and S2). SMP at baseline, its persistence after treatment, and plasmacytoma size ≥10 cm. were significant in univariate analysis for TTM. Multivariate analysis backwards selection model identified both SMP persistence at 1 year and plasmacytoma ≥10 cm. as independent variables for TTM rate with hazard ratios of 4.9 (95% CI 1.9–12.5; *p* = 0.001) and 4.5 (95% CI = 1.4–14.5; *p* < 0.012), respectively (Table S3 and Fig. S3). We used these two variables to create a model predicting the rate of progression to MM. Sixty-six patients with available data were included in this model (Table S4 and Fig. 1).

**Fig. 1:**
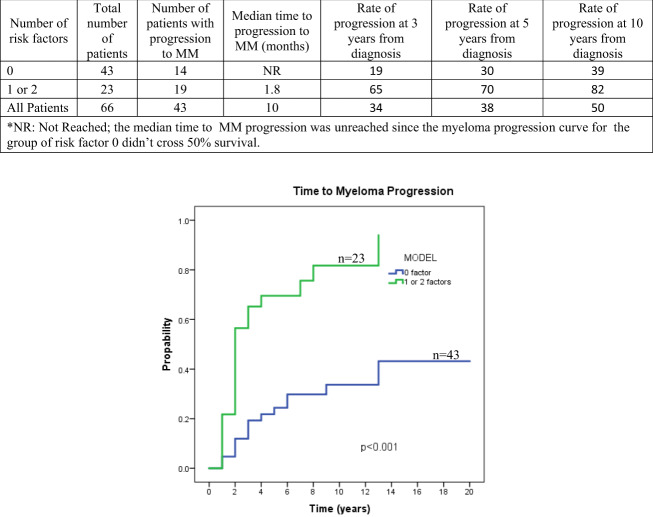
Model of high and low-risk SP by two risk factors (plasmacytoma size >10 cm. At diagnosis and persistent serum paraprotein 1 year after treatment) divided in to 2 groups: 0 risk factors and 1 and/or 2 risk factors. The rate of progression to MM was significantly higher in patients who had either a plasmacytoma size of ≥10 cm at diagnosis and/or persistent presence of serum paraprotein 1 year after treatment.

The median PFS for the entire cohort was 9.4 years (0.3–19.5 years). Baseline immunoparesis and presence of paraprotein at diagnosis and at 1 year after treatment were associated with unfavorable PFS. For these factors, the median PFS was 10 versus 6 years (*p* = 0.043), NR versus 4.5 years (*p* < 0.001), and NR versus 1.8 years (*p* < 0.001), respectively (Table S5/S6 and Fig. S3).

At the median follow-up time, 23 out of 71 patients (32%) had died (14/50 (28%) patients in SBP and 9/21 (42%) patients in SEP). In the SBP and SEP group, six (*n* = 6/14; 43%) vs three (*n* = 3/9; 33%) patients had a plasma cell disorder related death, respectively (*p* = 0.904). SBP patients were more likely to have a plasma cell disorder related death when compared to SEP patients. Because the cause of death was unknown in half of the patients: 35% (*n* = 5/14) in SBP and 55% (*n* = 5/9) in SEP, it is difficult to draw conclusions regarding this.

The median OS for the entire cohort was NR with 10-year OS of 76%. No OS difference was observed between SBP and SEP with a 10-year OS of 77 and 74% (*p* = 0.437), respectively. Patients with plasmacytoma size ≥10 cm had poorer OS (Table S7/S8 and Fig. S3).

Our study demonstrated comparable 3- and 5-year SP progression rates to MM with previous studies (14–38 and 45%, respectively) and confirmed that SBP patients have a shorter TTM than SEP patients. SBP tended to have worse PFS than SEP in our cohort, however it was not statistically significant (*p* = 0.263)^7–9^.

Several studies identified the presence of paraprotein at diagnosis, abnormal involved sFLC ratio, OMD, and residual paraprotein after treatment as poor prognosis factors for progression to MM^7,10,11^. Surprisingly, OMD did not predict progression in our cohort. This could be explained by flow analysis sensitivity in comparison to other published studies. For example, Paiva et al. used additional markers including CD20, CD27, CD28, CD81, and CD117 to determine clonality, which were not used in our analysis^7^. The impact of plasmacytoma size remains controversial. Our study found that patients with plasmacytomas ≥10 cm had unfavorable TTM, PFS, and OS. These patients may need treatment with higher RT doses for bulkier tumors and/or the addition of systemic therapy to local RT control.

This model (plasmacytoma >10 cm. and persistence of paraprotein 1 year after diagnosis) predicted, in our cohort, the progression risk to MM in SP patients (19% vs 65%, 30% vs 70%, and 39% vs 82% in patients with 0 and 1 or 2 factors, respectively). These rates are very similar (and even higher) than published rates of progression from studies for high-risk smoldering MM (SMM). Two randomized studies have shown improved PFS in SMM patients treated with lenalidomide ± dexamethasone^2,3^. One of these studies has also shown better OS for patients treated with lenalidomide/dexamethasone versus observation^3^. Adjuvant systemic therapy after radiation for SP patients to decrease the rate of progression to MM has been studied in the past. In 2019, a cooperative group study of 110 patients with SBP randomized to either ixazomib, lenalidomide, dexamethasone with zoledronic acid vs zoledronic acid (NCT02516423) after radiation therapy in the USA, was stopped due to lack of accrual. Thus, most studies investigating adjuvant therapy in SBP are either retrospective and/or in a small number of patients and the results remain controversial^12–14^. A recently published study of 61 SBP patients following a modern definition of plasmacytoma in France compared 37 patients treated with RT alone to 24 patients treated with the combination of RT and various adjuvant agents^15^. This study demonstrated a PFS benefit of the combination treatment, particularly in patients younger than 60 years of age.

Our study has several limitations including the retrospective nature of our data, no known cause of death for about half of the patients, inability to analyze SBP and EMP separately due to small EMP cohort size and absence of molecular/genetic analysis of plasmacytomas. Despite this, all patients underwent advanced imaging, BM MFC testing, and met IMWG 2014 criteria. We describe an easy to use clinical model including two variables: plasmacytoma >10 cm. at baseline and persistence of paraprotein after one year after treatment to predict progression to MM and may provide insights into the progression of patients in the clinic. These data need to be validated in other cohorts. International efforts are needed to lead adjuvant treatment studies in solitary plasmacytoma.

## Supplementary information

Supplementary Material
