# Supplementary material for: Serum paraprotein persistence and size determine outcome in a cohort of patients with a modern definition of plasmacytoma with up to 19 years of follow up
Source: Blood Cancer J. 2021 Feb 8;11(2):17. doi: 10.1038/s41408-021-00419-1 (PMC7873072; doi:10.1038/s41408-021-00419-1)
Supplement: Supplementary file 1 — Supplementary Material [file 41408_2021_419_MOESM1_ESM.docx]

**SUPPLEMENTARY MATERIAL**

# Serum paraprotein persistence and size determine outcome in a cohort of patients with a modern definition of plasmacytoma with up to 19 years of follow up

Manasanch et al., Department of Lymphoma/Myeloma, The University of Texas MD Anderson Cancer Center, Houston, Texas. 1515 Holcombe Blvd, Unit 429, Houston, TX 77030, USA.

**Table S1: Time to myeloma progression**

| **Factors** |  | **Number of patients** | **Number of events** | **Median TTM**  **(years)** | **3 year TTM**  **(%)** | **5 year TTM**  **(%)** | **10 year TTM**  **(%)** | **P value** |
| --- | --- | --- | --- | --- | --- | --- | --- | --- |
| **All cases** | **-** | 71 | 35 | 10 | 34 | 38 | 50 |  |
| **Bone** | **-** | 50 | 28 | 6.7 | 37 | 42 | 58 | 0.138 |
| **Extramedullary** | **-** | 21 | 7 | NR | 25 | 30 | 30 |  |
| **Immunoparesis** | **No** | 61 | 34 | NR | 29 | 34 | 43 | 0.124 |
|  | **Yes** | 6 | 6 | 6 | 50 | 50 | 100 |  |
| **Paraprotein at baseline** | **No** | 36 | 11 | NR | 23 | 26 | 30 | **0.002** |
|  | **Yes** | 30 | 19 | 5.3 | 41 | 49 | 63 |  |
| **Paraprotein at 1 year after treatment** | **No** | 42 | 14 | NR | 24 | 27 | 33 | **<0.001** |
|  | **Yes** | 19 | 16 | 1.8 | 68 | 74 | 82 |  |
| **Size >10cm** | **No** | 50 | 22 | NR | 37 | 42 | 45 | **0.033** |
|  | **Yes** | 6 | 5 | 1.5 | 67 | 67 | 83 |  |
| **Occult marrow disease (Bone marrow aspirate positive for aberrant plasma cells by flow cytometry)** | **No** | 64 | 34 | 7 | 41 | 47 | 57 | 0.067 |
| **RT 45 Gy** | **No** | 15 | 6 | NR | 41 | 41 | 41 | 0.464 |
|  | **Yes** | 52 | 29 | 8 | 37 | 43 | 53 |  |
| Abbreviations: RT, radiation; TTM , time to myeloma progression | | | | | | | | |

| **Factors** |  | **Number of patients** | | **Number of events** | | **Median TTM**  **(years)** | | **3 year TTM**  **(%)** | | **5 year TTM**  **(%)** | | **10 year TTM**  **(%)** | | **P value** |
| --- | --- | --- | --- | --- | --- | --- | --- | --- | --- | --- | --- | --- | --- | --- |
|  |  | **B** | **E** | **B** | **E** | **B** | **E** | **B** | **E** | **B** | **E** | **B** | **E** |  |
| **Immunoparesis** | **No** | 42 | 19 | 21 | 6 | 8.9 | NR | 35 | 28 | 43 | 28 | 56 | 28 | 0.182 |
|  | **Yes** | 4 | 2 | 3 | 1 | 6 | 3 | 50 | 50 | 50 | 100 | 100 | 100 |  |
| **Paraprotein at baseline** | **No** | 19 | 17 | 6 | 5 | NR | NR | 27 | 25 | 27 | 25 | 36 | 25 | **0.008** |
|  | **Yes** | 26 | 4 | 17 | 2 | 5.4 | 6 | 44 | 50 | 53 | 50 | 73 | 50 |  |
| **Paraprotein at 1 year after treatment** | **No** | 26 | 16 | 9 | 5 | 18 | NR | 24 | 25 | 32 | 25 | 38 | 25 | **<0.001** |
|  | **Yes** | 18 | 1 | 15 | 1 | 2 | 0.8 | 72 | 100 | 72 | 100 | 19 | 100 |  |
| **Size >10cm** | **No** | 36 | 14 | 17 | 5 | 12.3 | NR | 38 | 36 | 45 | 36 | 49 | 36 | 0.058 |
|  | **Yes** | 5 | 1 | 4 | 1 | 2 | 0.8 | 60 | 100 | 60 | NA | 80 | NA |  |
| **Occult marrow disease** | **No** | 44 | 20 | 27 | 7 | 5.4 | 19 | 45 | 32 | 53 | 32 | 79 | 32 | 0.43 |
|  | **Yes** | 6 | 1 | 1 | 0 | 13 | 8 | 0 | 0 | 0 | 0 | 22 | NA |  |
| **RT 45 Gy** | **< 45 Gy** | 9 | 6 | 4 | 2 | 15 | NR | 44 | 35 | 44 | 35 | 44 | 35 | 0.662 |
|  | **≥ 45 Gy** | 40 | 13 | 24 | 5 | 6 | NR | 39 | 31 | 47 | 31 | 66 | 31 |  |

**Table S2: Time to myeloma progression divided by bone or extra-medullary plasmacytoma**

| Abbreviations: B, solitary bone plasmacytoma; E, solitary extramedullary plasmacytoma; RT, radiation; TTM , time to myeloma progression |
| --- |

**Table S3: Multivariate analysis for time to myeloma progression, progression free survival, and overall survival**

| **Factors** | **TTM** | | **Factors** | **PFS** | | **Factors** | **OS** | |
| --- | --- | --- | --- | --- | --- | --- | --- | --- |
|  | **Hazard ration**  **(95%CI)** | **p-value** |  | **Hazard ration**  **(95%CI)** | **p-value** |  | **Hazard ration**  **(95%CI)** | **p-value** |
| **Size ≥10cm.** | 4.5 (1.3 – 14) | **0.012** | **Immunoparesis** | 4.1 (1.3 – 13) | **0.017** | **Size ≥10cm.** | 3.5 (1.1 – 11) | **0.032** |
| **Paraprotein at 1 year** | 4.9 (2 – 12) | **<0.001** | **Paraprotein at 1 year** | 3.6 (1.5 – 8.4) | **0.003** | - | - | - |
| Abbreviations: OS, overall survival; PFS, progression free survival; TTM , time to myeloma progression | | | | | | | | |

**Table S4: Clinical model of progression using two risk factors (immunoparesis at diagnosis and persistent serum paraprotein after one year of treatment) in SBP and SEP patients with TTM, PFS and OS.**

| **Number of risk factors**  **SBP** | **Total patients** | **Number of events** | **Median TTM (years)** | **p-value** | **Number of events** | **Median PFS (years)** | **p-value** | **Number of events** | **Median OS (years)** | **p-value** |
| --- | --- | --- | --- | --- | --- | --- | --- | --- | --- | --- |
| **0** | 27 | 9 | NR | **<0.001** | 12 | 8 | **0.001** | 5 | NR | 0.048 |
| **1 or 2** | 21 | 17 | 2 |  | 17 | 2 |  | 8 | 12.7 |  |
| **All Patients** | 48 | 26 | 6.7 |  | 29 | 6.3 |  | 10 | NR |  |
| **Number of risk factors**  **SEP** | **Total patients** | **Number of events** | **Median TTM (years)** | **p-value** | **Number of events** | **Median PFS (years)** | **p-value** | **Number of events** | **Median OS (years)** | **p-value** |
| **0** | 16 | 5 | NR | **0.01** | 8 | NR | **0.01** | 7 | NR | 0.584 |
| **1 or 2** | 2 | 2 | 0.8 |  | 2 | 0.8 |  | 1 | 11 |  |
| **All Patients** | 18 | 7 | NR |  | 10 | 11 |  | 8 | 16 |  |
| *NR: Not reached; the median time was not reached since the survival curve for the group risk factor didn’t cross 50% survival.  Abbreviations: SBP, solitary bone plasmacytoma; SEP, solitary extramedullary plasmacytoma | | | | | | | | | | |

**Table S5: Progression free survival for the entire cohort of patients**

| **Factors** |  | **Number of patients** | **Number of events** | **Median PFS**  **(years)** | **3 year PFS**  **(%)** | **5 year PFS**  **(%)** | **10 year PFS**  **(%)** | **P value** |
| --- | --- | --- | --- | --- | --- | --- | --- | --- |
| **All cases** | **-** | 71 | 42 | 6.9 | 61 | 56 | 40 |  |
| **Bone** | **-** | 50 | 31 | 7 | 59 | 59 | 32 | 0.263 |
| **Extramedullary** | **-** | 21 | 11 | 12 | 65 | 65 | 59 |  |
| **Immunoparesis** | **No** | 61 | 33 | 10 | 64 | 59 | 47 | **0.043** |
|  | **Yes** | 6 | 5 | 6 | 50 | 50 | 0 |  |
| **Presence of paraprotein at baseline** | **No** | 36 | 15 | NR | 71 | 71 | 64 | **<0.001** |
|  | **Yes** | 30 | 22 | 5 | 52 | 44 | 19 |  |
| **Paraprotein persistence at 1 year after treatment** | **No** | 42 | 20 | 12 | 74 | 69 | 57 | **<0.001** |
|  | **Yes** | 19 | 16 | 1.8 | 26 | 26 | 18 |  |
| **Size >10cm** | **No** | 50 | 27 | 6.8 | 61 | 56 | 45 | 0.105 |
|  | **Yes** | 6 | 5 | 1.5 | 33 | 33 | 17 |  |
| **Occult marrow disease** | **No** | 66 | 41 | 6 | 56 | 51 | 36 | **0.037** |
|  | **Yes** | 7 | 1 | 13 | 100 | 100 | 82 |  |
| **RT 45 Gy** | **< 45 Gy** | 22 | 7 | 11 | 59 | 59 | 59 | 0.336 |
|  | **≥ 45 Gy** | 67 | 35 | 6 | 60 | 44 | 19 |  |

Abbreviations: RT, radiation; PFS, progression free survival

**Table S6: Progression free survival divided by bone or extramedullary plasmacytoma**

|  |  | **Number of patients** | | **Number of events** | | **Median PFS**  **(years)** | | **3 year PFS**  **(%)** | | **5 year PFS**  **(%)** | | **10 year PFS**  **(%)** | | **P value** |
| --- | --- | --- | --- | --- | --- | --- | --- | --- | --- | --- | --- | --- | --- | --- |
|  |  | **B** | **E** | **B** | **E** | **B** | **E** | **B** | **E** | **B** | **E** | **B** | **E** |  |
| **Immunoparesis** | **No** | 42 | 19 | 23 | 19 | 8 | 12 | 63 | 67 | 55 | 67 | 40 | 61 | **0.076** |
|  | **Yes** | 4 | 2 | 4 | 2 | 6 | 3 | 50 | 50 | 50 | 50 | 0 | NA |  |
| **Paraprotein** | **No** | 19 | 17 | 8 | 7 | NR | NR | 68 | 75 | 68 | 75 | 55 | 75 | **0.003** |
|  | **Yes** | 26 | 4 | 18 | 4 | 5 | 2 | 56 | 25 | 47 | 25 | 23 | 0 |  |
| **Paraprotein at 1 year after treatment** | **No** | 26 | 16 | 12 | 8 | NR | 12 | 73 | 75 | 65 | 75 | 51 | 68 | **<0.001** |
|  | **Yes** | 18 | 1 | 15 | 1 | 1.7 | 0.8 | 33 | 0 | 28 | 0 | 19 | 0 |  |
| **Size >10cm** | **No** | 36 | 14 | 20 | 7 | 6.3 | 11.2 | 60 | 64 | 53 | 55 | 41 | 55 | 0.114 |
|  | **Yes** | 5 | 1 | 4 | 1 | 2 | 0.8 | 40 | 0 | 40 | 0 | 20 | 0 |  |
| **Occult marrow disease** | **No** | 44 | 20 | 30 | 11 | 5 | 11.7 | 56 | 69 | 46 | 63 | 26 | 57 |  |
|  | **Yes** | 6 | 1 | 1 | 0 | 13 | 8 | 100 | 100 | 100 | 199 | 78 | 100 | **0.025** |
| **RT 45 Gy** | **< 45 Gy** | 9 | 6 | 4 | 3 | Nr | 11 | 56 | 65 | 56 | 65 | 56 | 65 |  |
|  | **≥ 45 Gy** | 40 | 13 | 27 | 8 | 6 | 12 | 59 | 62 | 51 | 62 | 33 | 54 | 0.457 |

Abbreviations: B, solitary bone plasmacytoma; E, solitary extramedullary plasmacytoma; RT, radiation; PFS, progression free survival

**Table S7: Overall Survival**

| **Factors** |  | **Number of patients** | **Number of events** | **Median OS** | **3 year OS**  **(%)** | **5 year OS**  **(%)** | **10 year OS**  **(%)** | **P value** |
| --- | --- | --- | --- | --- | --- | --- | --- | --- |
| **All cases** | **-** | 71 | 23 | 6.7 | 94 | 91 | 76 | - |
| **Bone** | **-** | 50 | 14 | 6 | 59 | 52 | 32 | 0.437 |
| **Extra bone** | **-** | 21 | 9 | 12 | 65 | 65 | 59 |  |
| **Immunoparesis** | **No** | 61 | 17 | 10 | 64 | 59 | 50 | 0.259 |
|  | **Yes** | 6 | 3 | 6 | 50 | 50 | 0 |  |
| **Paraprotein** | **No** | 36 | 12 | 18 | 71 | 71 | 64 | 0.986 |
|  | **Yes** | 30 | 8 | 4.5 | 52 | 44 | 19 |  |
| **Paraprotein at 1 year after treatment** | **No** | 42 | 13 | 12 | 74 | 69 | 57 | 0.492 |
|  | **Yes** | 19 | 6 | 1.8 | 26 | 26 | 18 |  |
| **Size >10cm** | **No** | 50 | 13 | 6.8 | 61 | 56 | 45 | **0.022** |
|  | **Yes** | 6 | 4 | 1.5 | 33 | 33 | 17 |  |
| **Occult marrow disease** | **No** | 66 | 23 | 6 | 56 | 51 | 36 | 0.078 |
|  | **Yes** | 7 | 0 | NR | 100 | 100 | 82 |  |
| **RT 45 Gy** | **< 45 Gy** | 22 | 4 | 11 | 59 | 59 | 59 | 0.890 |
|  | **≥ 45 Gy** | 67 | 19 | 6 | 60 | 54 | 35 |  |
| Abbreviations: RT, radiation; OS, overall survival | | | | | | | | |

**Table S8: Overall survival divided by bone or extramedullary plasmacytoma**

|  |  | **Number of patients** | | **Number of events** | | **Median OS**  **(years)** | | **3 year OS**  **(%)** | | **5 year OS**  **(%)** | | **10 year OS (%)** | | **P value** |
| --- | --- | --- | --- | --- | --- | --- | --- | --- | --- | --- | --- | --- | --- | --- |
|  |  | **B** | **E** | **B** | **E** | **B** | **E** | **B** | **E** | **B** | **E** | **B** | **E** |  |
| **Immunoparesis** | **No** | 42 | 19 | 9 | 8 | NR | 12.6 | 84 | 75 | 84 | 75 | 64 | 69 | 0.147 |
|  | **Yes** | 4 | 2 | 2 | 1 | 6 | 3 | 75 | 50 | 75 | 0 | 0 | 0 |  |
| **Paraprotein** | **No** | 19 | 17 | 5 | 7 | NR | NR | 84 | 75 | 84 | 74 | 67 | 75 | 0.649 |
|  | **Yes** | 26 | 4 | 6 | 2 | NR | 6 | 82 | 100 | 82 | 50 | 51 | 0 |  |
| **Paraprotein at 1 year after treatment** | **No** | 26 | 16 | 6 | 7 | NR | 12 | 88 | 80 | 88 | 80 | 68 | 72 | 0.357 |
|  | **Yes** | 18 | 1 | 6 | 0 | NR | 0.6 | 59 | 0 | 59 | 0 | 59 | 0 |  |
| **Size >10cm** | **No** | 36 | 14 | 8 | 5 | NR | 11.3 | 81 | 75 | 81 | 75 | 67 | 64 | **0.004** |
|  | **Yes** | 5 | 1 | 3 | 1 | NR | 0.8 | 57 | 0 | 57 | 0 | 29 | 0 |  |
| **Occult marrow disease** | **No** | 44 | 20 | 14 | 9 | 10 | 12 | 76 | 100 | 76 | 100 | 55 | 100 | 0.096 |
|  | **Yes** | 6 | 1 | 0 | 0 | 13 | NR | 71 | 100 | 71 | 100 | 64 | 100 |  |
| **RT 45 Gy** | **< 45 Gy** | 9 | 6 | 1 | 3 | NR | 11 | 88 | 78 | 88 | 78 | 88 | 57 | 0.684 |
|  | **≥ 45 Gy** | 40 | 13 | 13 | 6 | 10 | 13 | 65 | 74 | 65 | 74 | 65 | 65 |  |
| Abbreviations: B, solitary bone plasmacytoma; E, solitary extramedullary plasmacytoma; RT, radiation; OS, overall survival | | | | | | | | | | | | | | |

**Figure S1: Study flow**

**Figure S2: Time to myeloma progression based on A) Bone and extra-medullary plasmacytoma, B) Serum paraprotein presence at diagnosis C) Persistent serum paraprotein at 1 year after diagnosis, and D) plasmacytoma size > 10 cm at diagnosis**


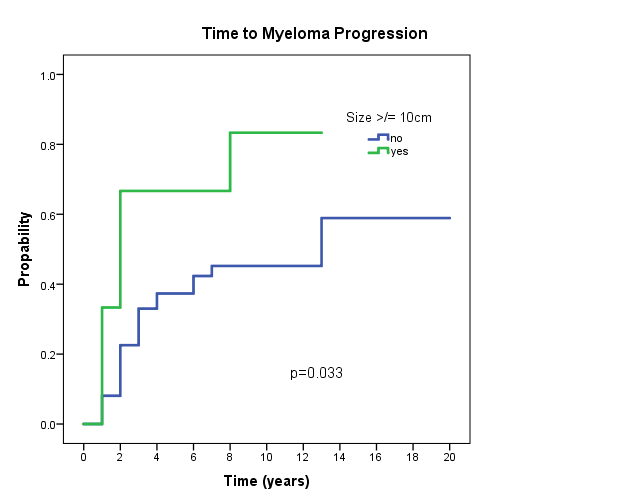

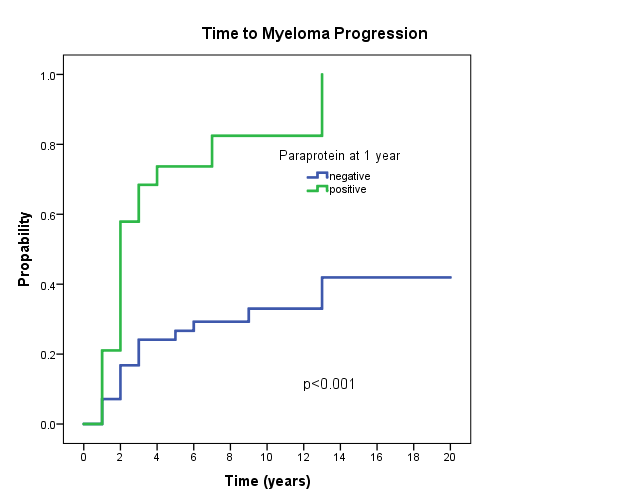

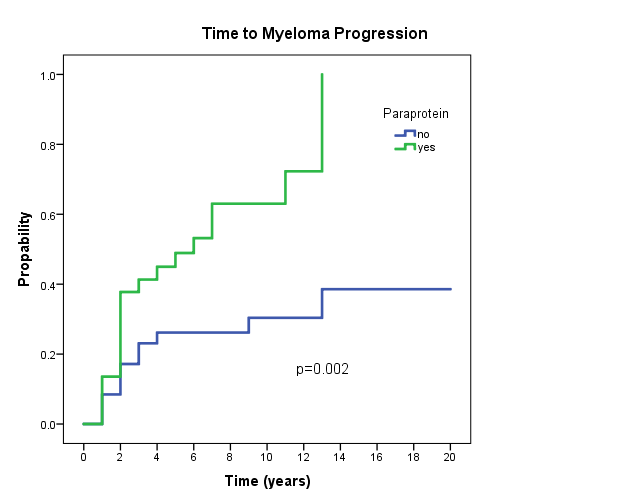

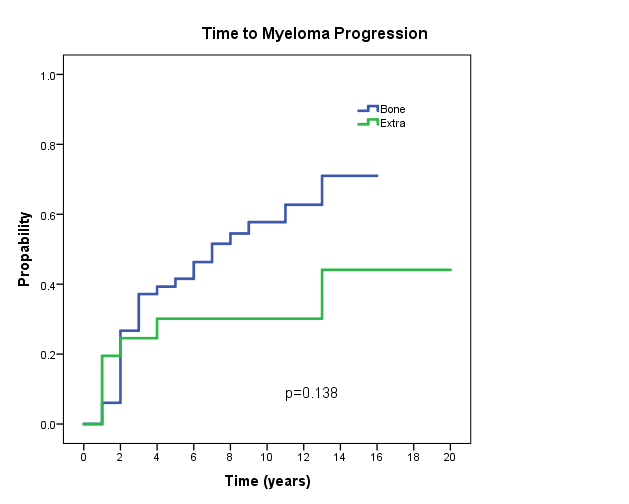
**Figure S3: Progression free survival (A and B) and overall survival (C and D) based on bone and extramedullary plasmacytoma and presence of serum paraprotein one year after diagnosis.**

n=50

n=6

n=42

n=19

n=39

n=32

n=21

n=50

**D**

**C**

**B**

**A**

n=21

**
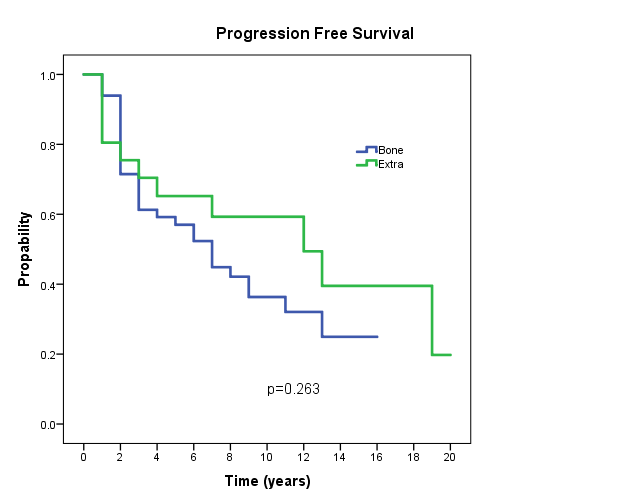
**
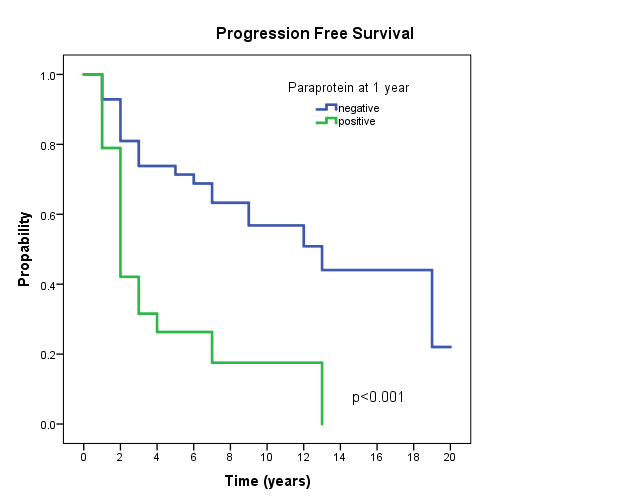


**A**

**B**


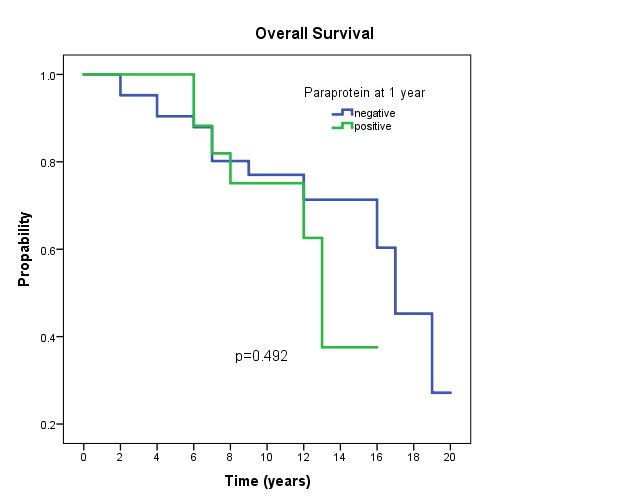

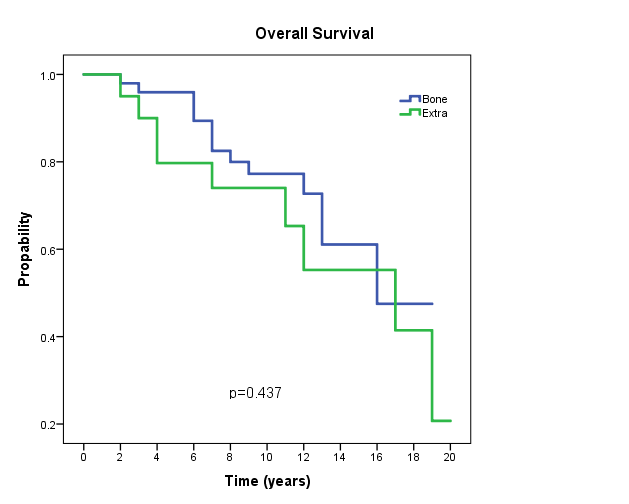


n=21

n=50

n=19

n=19

n=42

n=42

n=50

**D**

**C**
